# Supplementary material for: Simultaneous Copy Number Alteration and Single-Nucleotide Variation Analysis in Matched Aqueous Humor and Tumor Samples in Children with Retinoblastoma
Source: Int J Mol Sci. 2023 May 11;24(10):8606. doi: 10.3390/ijms24108606 (PMC10218537; doi:10.3390/ijms24108606)
Supplement: Supplementary file 1 [file ijms-24-08606-s001.zip › supplementary_figures.pdf]

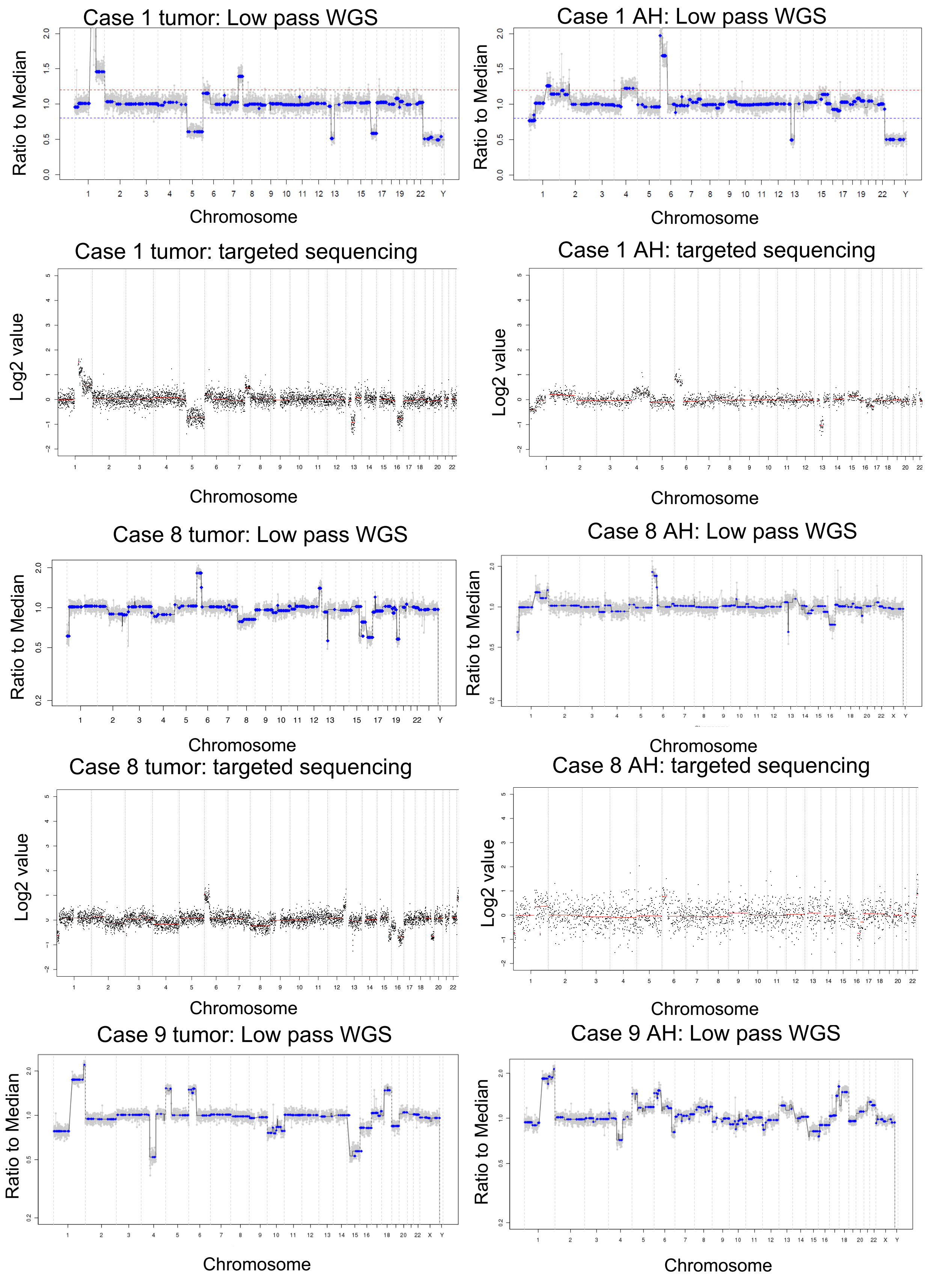

Case 1 tumor: Low pass WGS
Ratio to Median
Chromosome
Case 1 AH: Low pass WGS
Ratio to Median
Chromosome
Case 1 tumor: targeted sequencing
Log2 value
Chromosome
Case 1 AH: targeted sequencing
Log2 value
Chromosome
Case 8 tumor: Low pass WGS
Ratio to Median
Chromosome
Case 8 AH: Low pass WGS
Ratio to Median
Chromosome
Case 8 tumor: targeted sequencing
Log2 value
Chromosome
Case 8 AH: targeted sequencing
Log2 value
Chromosome
Case 9 tumor: Low pass WGS
Ratio to Median
Chromosome
Case 9 AH: Low pass WGS
Ratio to Median
Chromosome

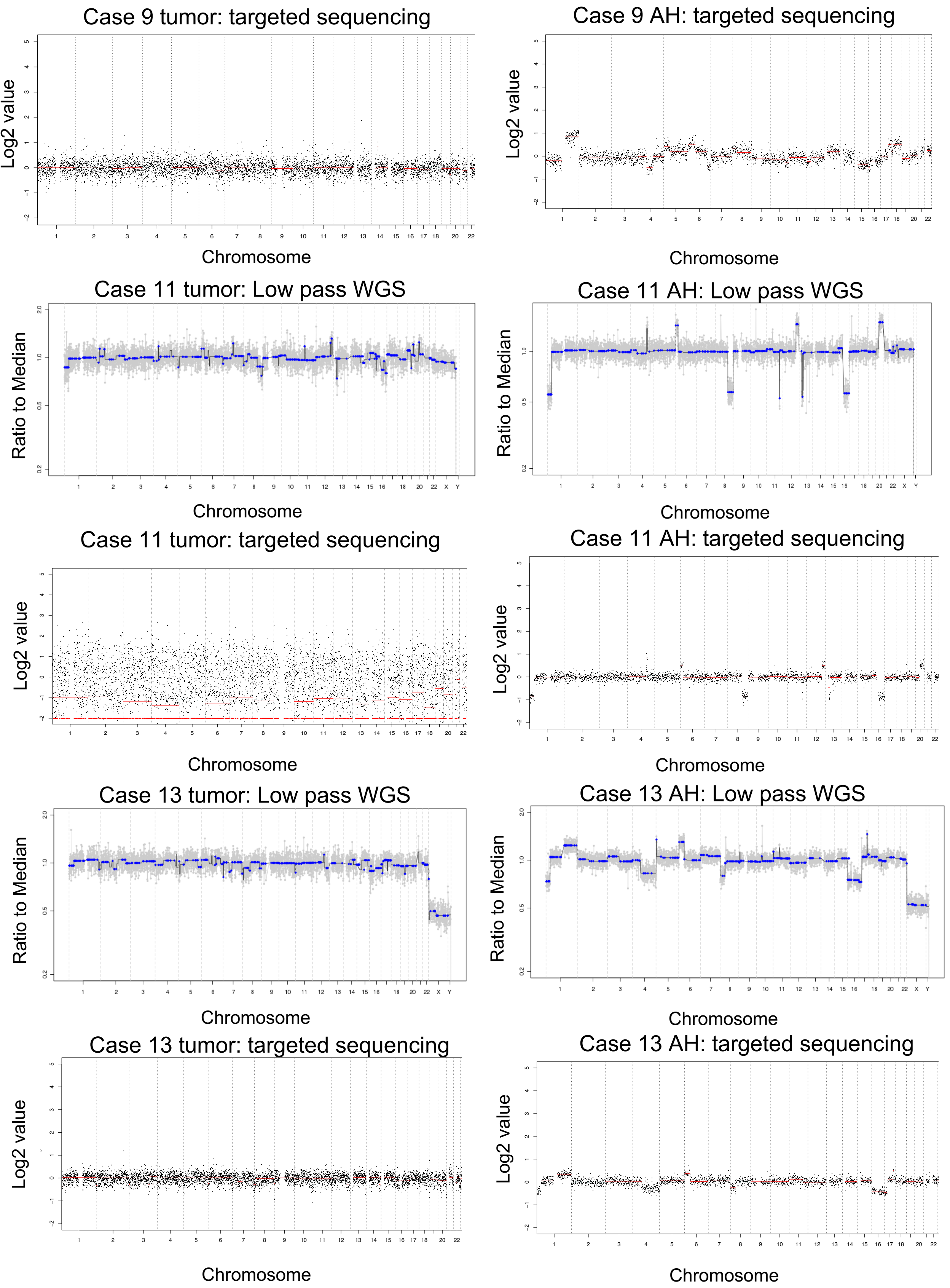

Case 9 tumor: targeted sequencing
Log2 value
Chromosome
Case 9 AH: targeted sequencing
Log2 value
Chromosome
Case 11 tumor: Low pass WGS
Ratio to Median
Chromosome
Case 11 AH: Low pass WGS
Ratio to Median
Chromosome
Case 11 tumor: targeted sequencing
Log2 value
Chromosome
Case 11 AH: targeted sequencing
Log2 value
Chromosome
Case 13 tumor: Low pass WGS
Ratio to Median
Chromosome
Case 13 AH: Low pass WGS
Ratio to Median
Chromosome
Case 13 tumor: targeted sequencing
Log2 value
Chromosome
Case 13 AH: targeted sequencing
Log2 value
Chromosome

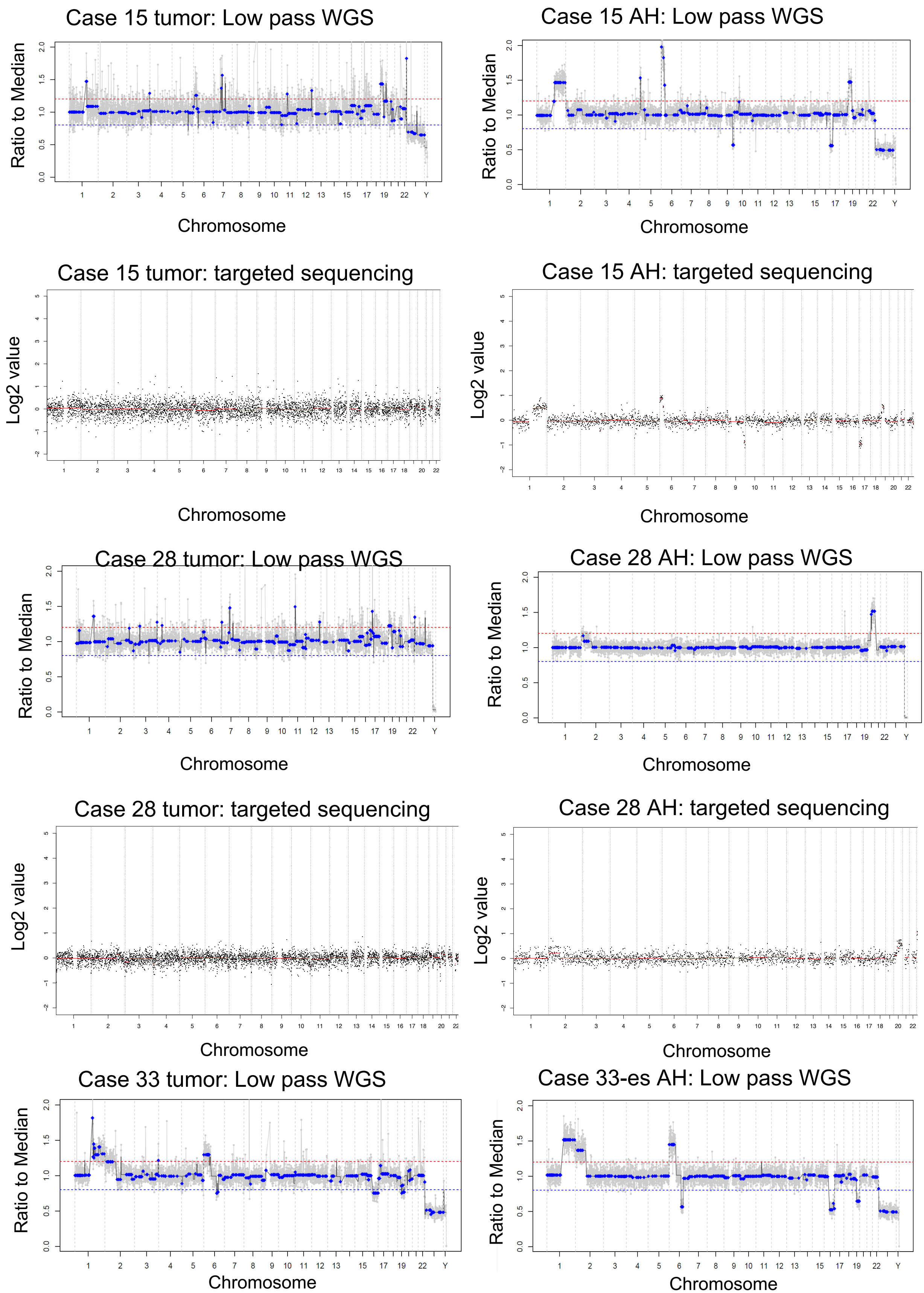

Case 15 tumor: Low pass WGS
Ratio to Median
Chromosome
Case 15 AH: Low pass WGS
Ratio to Median
Chromosome
Case 15 tumor: targeted sequencing
Log2 value
Chromosome
Case 15 AH: targeted sequencing
Log2 value
Chromosome
Case 28 tumor: Low pass WGS
Ratio to Median
Chromosome
Case 28 AH: Low pass WGS
Ratio to Median
Chromosome
Case 28 tumor: targeted sequencing
Log2 value
Chromosome
Case 28 AH: targeted sequencing
Log2 value
Chromosome
Case 33 tumor: Low pass WGS
Ratio to Median
Chromosome
Case 33-es AH: Low pass WGS
Ratio to Median
Chromosome

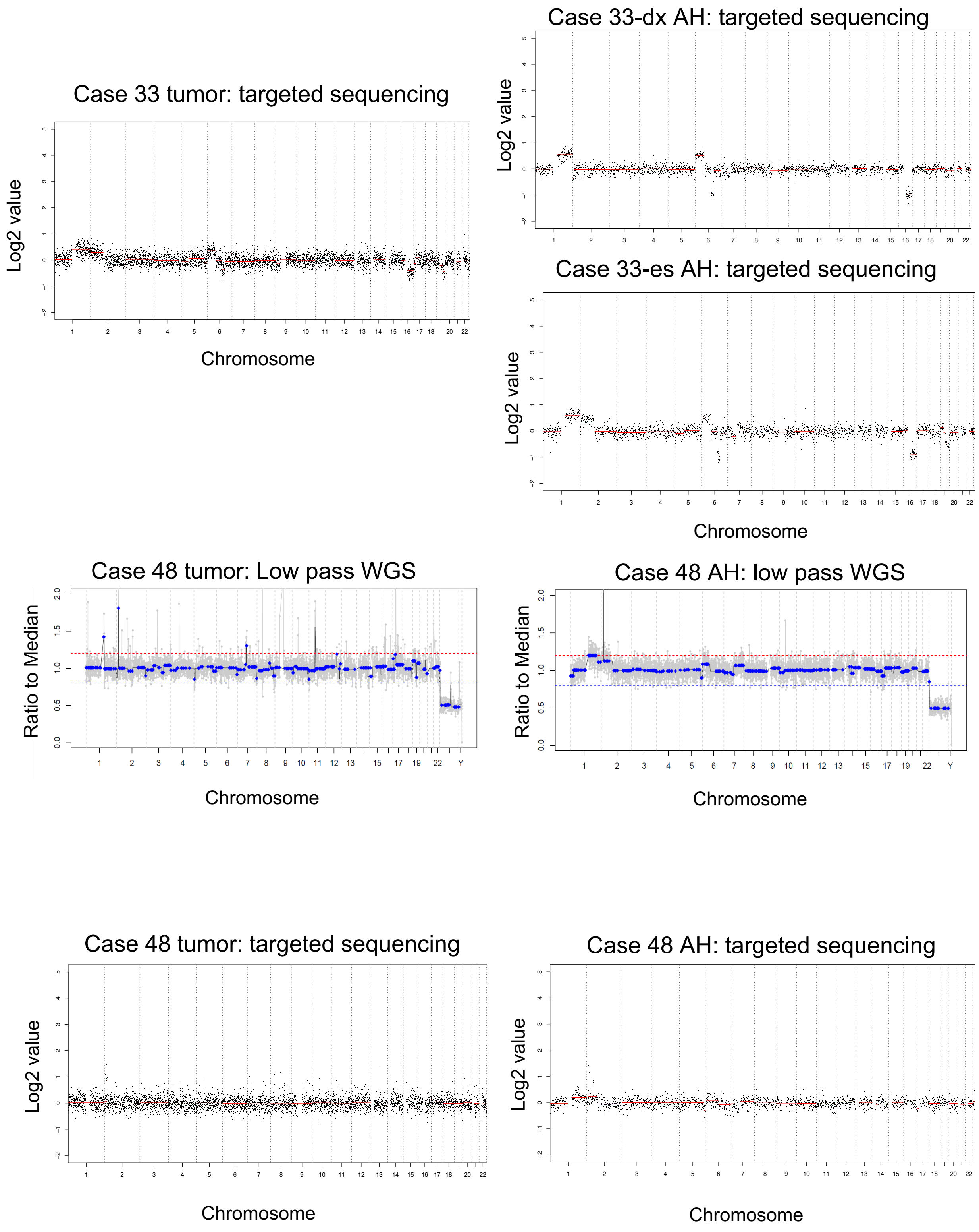
Case 33 tumor: targeted sequencing
Log2 value
Chromosome
Case 33-dx AH: targeted sequencing
Log2 value
Case 33-es AH: targeted sequencing
Log2 value
Chromosome
Case 48 tumor: Low pass WGS
Ratio to Median
Chromosome
Case 48 AH: low pass WGS
Ratio to Median
Chromosome
Case 48 tumor: targeted sequencing
Log2 value
Chromosome
Case 48 AH: targeted sequencing
Log2 value
Chromosome

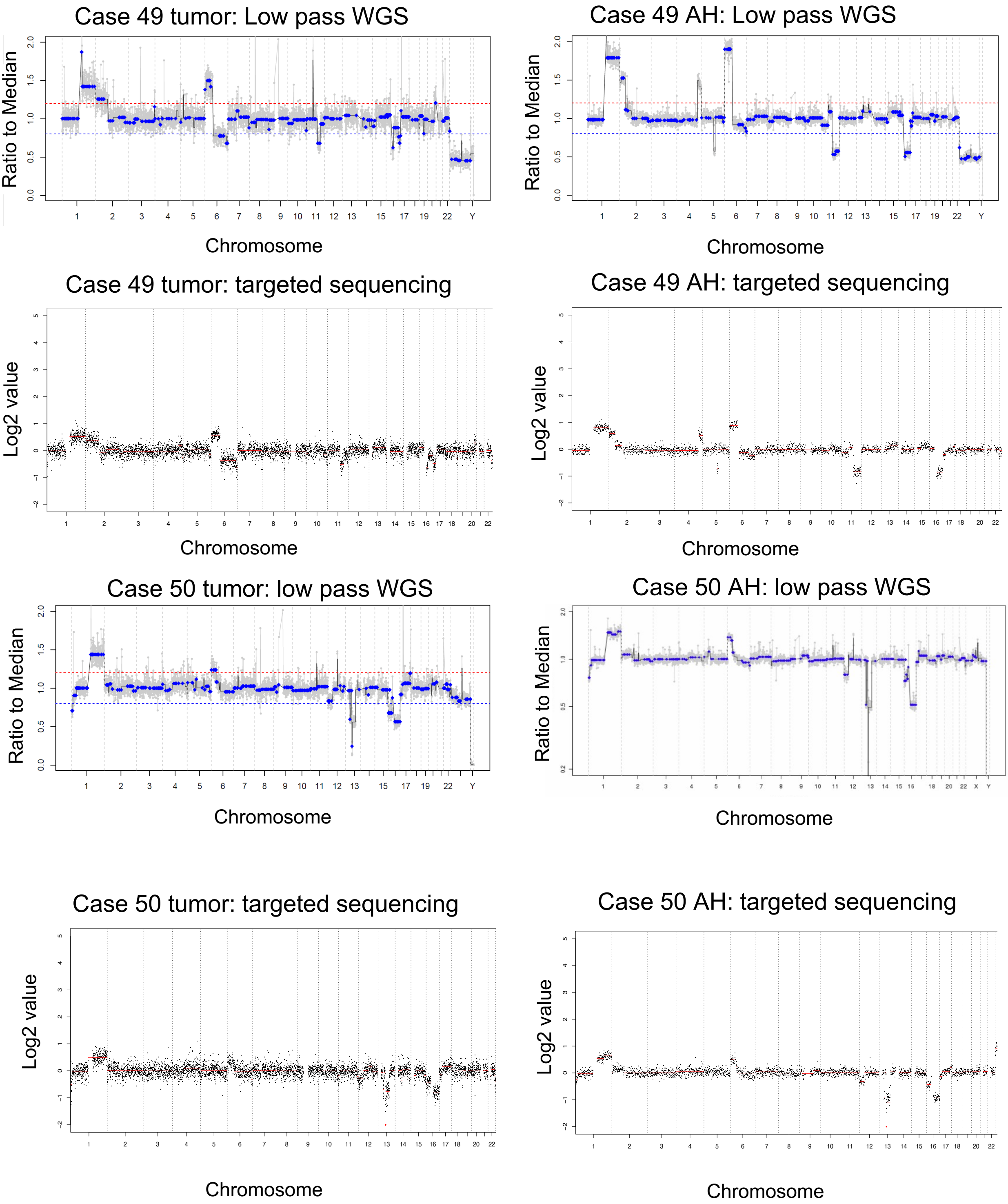

Figure S1: SCNA profiles for low pass WGS and targeted sequencing samples for every AH and tumor sample in the cohort. Low pass WGS plots were generated with internal pipeline, while targeted sequencing plots were made with CopyWriteR. CopyWriteR utilized a female normal control; therefore, sex chromosomes were not considered for analysis (only chromosomes 1-22).

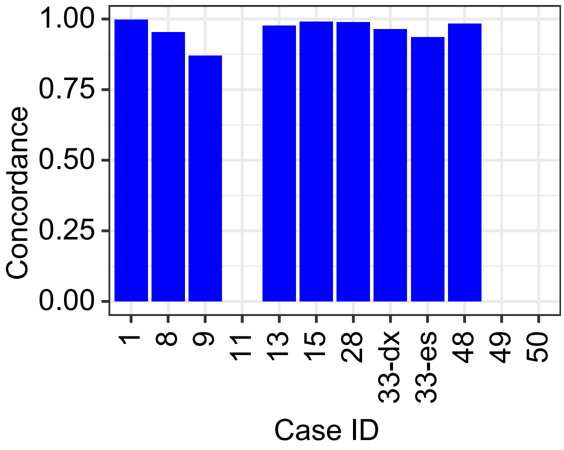

Figure S2: Concordance for targeted AH to low pass WGS AH samples. Samples 11, 49, and 50 were sequenced single-end 50bp (remaining patients sequenced paired-end 150bp) and concordances calculations were deemed to not be comparable for these patients

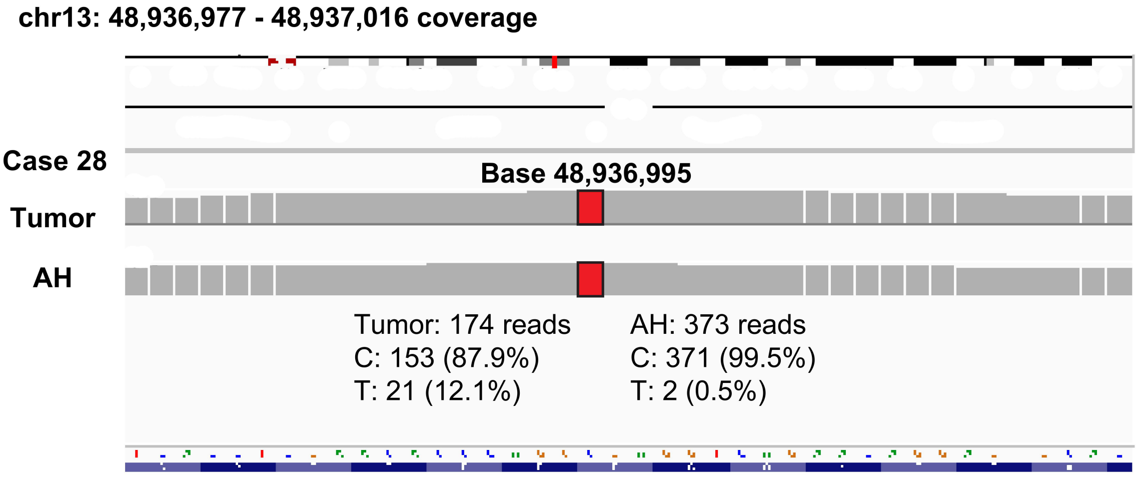

Figure S3: IGV snapshot for case 28 displaying p.R255* variant in the tumor but not the AH
